# Adverse pregnancy outcomes, longitudinal change in eGFR, and incident hypertension in women: a population-based cohort study

**DOI:** 10.1080/0886022X.2026.2644784

**Published:** 2026-04-29

**Authors:** Marzieh Saei Ghare Naz, Maryam Mousavi, Maryam Farahmand, Mahsa Noroozzadeh, Fereidoun Azizi, Fahimeh Ramezani Tehrani

**Affiliations:** a Reproductive Endocrinology Research Center, Research Institute for Endocrine Molecular Biology, Research Institute for Endocrine Sciences, Shahid Beheshti University of Medical Sciences, Tehran, Iran; b Endocrine Research Center, Research Institute for Endocrine Disorders, Research Institute for Endocrine Sciences, Shahid Beheshti University of Medical Sciences, Tehran, Iran; c Foundation for Research and Education Excellence, Vestavia Hills, AL, USA

**Keywords:** Adverse pregnancy outcomes, estimated glomerular filtration rate, hypertension incidence, renal function trajectory, women’s cardiovascular health, population-based cohort

## Abstract

**Background:**

As the traditional risk factors cannot account for all hypertension (HTN) incidents, it is of great importance to determine other risk factors. This study aimed to identify long-term HTN risk associated with annual change of estimated glomerular filtration rate (eGFR) and prior adverse pregnancy outcomes (APOs) among women participating in a population-based study of Tehran Lipid and Glucose Study (TLGS).

**Methods:**

This study was performed using prospectively ascertained data of TLGS. A total of 2,404 women with recorded data of eGFR measurements and APO status participated. Data collection was conducted according to the standard guide of TLGS. Cox proportional-hazards regression models were used to estimate the hazard ratios (HRs) and their 95% confidence intervals (CIs) for the incidence of HTN.

**Results:**

A total of 2,404 women were enrolled. Adjusted model shows that, a one z-score positive eGFR change was associated with a reduced risk of developing HTN among women with a history of APOs (HR= 0.816, 95% CI: 0.708–0.939, *p* = 0.005). In contrast, no significant association was observed among women without a history of APOs (HR= 1.027, 95% CI: 0.913–1.156, *p* = 0.645). We also observed a statistically significant interaction between APO status and annual eGFR change for HTN incidence in total population (interaction *p* = 0.02).

**Conclusion:**

The history of APOs is accompanied by alterations in kidney function in the long term. In women with a history of APOs, a positive change in eGFR levels was independently associated with a lower risk of HTN.

## Graphical Abstract

**Figure:**
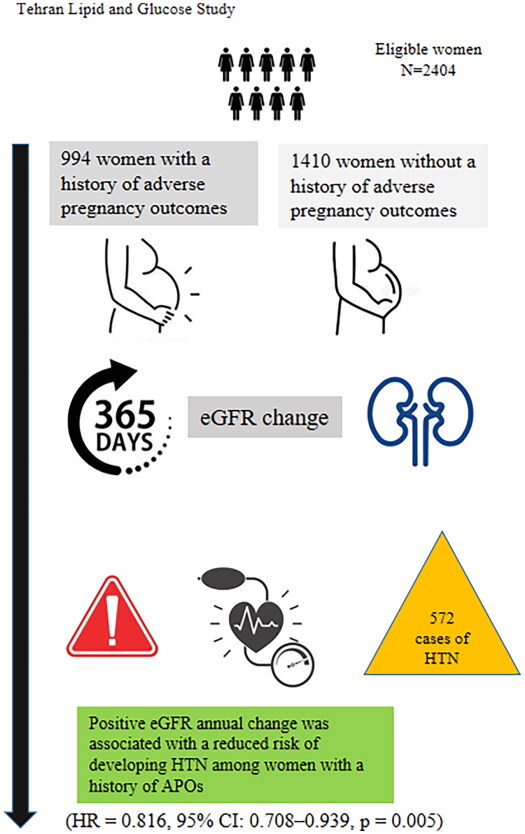

**Clinical implications**
- Women with a history of adverse pregnancy outcomes represent a distinct high-risk group for future hypertension and warrant targeted long-term follow-up.
- Longitudinal changes in estimated glomerular filtration rate provide clinically relevant prognostic information beyond a single baseline renal function measurement.
- Preserved or improving eGFR trajectories may identify women with prior adverse pregnancy outcomes who are at lower subsequent risk of developing hypertension.
- Routine assessment of renal function trajectories could enhance early hypertension risk stratification in women with complicated pregnancy histories.
- Pregnancy history should be systematically integrated into cardiovascular and renal risk assessment algorithms for women.
- Postpartum and midlife surveillance strategies may benefit from incorporating serial eGFR monitoring, particularly after adverse pregnancy outcomes.
- Early identification of declining renal function may enable timely lifestyle or pharmacological interventions to prevent hypertension.
- These findings support a life-course approach to women’s health, linking reproductive events to long-term cardio-renal outcomes.
- Collaboration between obstetrics, primary care, nephrology, and cardiology is essential for optimizing preventive care in this population.
- Recognizing pregnancy as a physiological stress test offers an opportunity to implement precision prevention strategies for future hypertension.

## Introduction

The rising trend of hypertension (HTN) in recent years has made it a substantial public health concern [1]. Evidence supported that a wide range of factors, including genetic, environmental, lifestyle, metabolic components and reproductive factors, ethnicity, education, and socioeconomic level, contribute to the development of HTN [1–7]. It is reported that approximately one quarter of Iranian adults suffer from HTN [8]. HTN is known as a major risk factor for cardiovascular disease (CVDs) [9].

Women with a history of adverse pregnancy outcome (APO) have been shown to be at increased risk for the development of HTN later in life [10]. It is reported that one in five live births is complicated by APOs [11]. There is evidence showing that women, after experiencing adverse pregnancy outcomes, are prone to kidney function deterioration [12]. Interestingly, a recent study reported that women with a history of APOs are predisposed to chronic kidney disease up to 46 years later [13].

At the same time, estimated glomerular filtration rate (eGFR) slope is also known as a predictor of cardiovascular disease [14]. However, kidney dysfunction is the most common cause of HTN [15]. Han et al. (2023) reported a reduced eGFR impact on the incidence metabolic syndrome [16]. Another study among the Japanese Population without CKD showed that there is a significant association with eGFR decline and metabolic syndrome [17]. In Chinese hypertensive patients, both the decline and increase of eGFR levels were independently associated with the risks of first stroke or first ischemic stroke [18]. It is proposed that women who experience APOs have been reported to have an elevated risk of kidney-related complications [19].

The physiological state of pregnancy is associated with an alteration of serum creatinine concentration not only during pregnancy but also after pregnancy [20]. Pregnancy complications are recognized as lifelong risk factors for the development of HTN and mortality in women [21,22]. Literature on the association of exposure with APOs and eGFR changes with the development of HTN has focused mainly on the separate association of APOs and later kidney function [23] and APOs and later development of HTN [24] as well as the association of kidney function with HTN [15]. Understanding the potential relationship between exposure to change of eGFR and development of HTN in women stratified by history of APOs is crucial for identifying high-risk women to mitigate long-term HTN risk. Therefore, this study aimed to identify long-term HTN risk associated with eGFR changes and APOs among women who participated in a population-based study of Tehran Lipid and Glucose Study (TLGS).

## Methods

### Study design and participants

This study was performed using prospectively ascertained data of Tehran Lipid and Glucose Study (TLGS), a population-based cohort with 20 years of follow-up, initiated in 1998 to investigate the prevalence and risk factors of non-communicable diseases. Details of this cohort have been described elsewhere [25]. The follow-up visits were performed every 3 years to obtain data on demographics, anthropometric, reproductive, hormonal, and metabolic characteristics, general physical examinations, and laboratory measurements.

**Figure 1.**
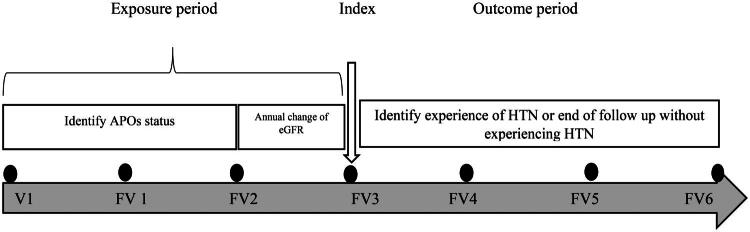
Diagram of study exposure and outcome variables measurement. **Abbreviation**: V: visit; FV: follow-up visit; APO; adverse pregnancy outcome; eGFR; estimated glomerular filtration rate.

Of the 4,708 women, those with missing data in annual change of eGFR (*n* = 634), women with eGFR˂15 mL/min/1.73 m^2^ (*n* = 1), women with a history of HTN before third follow-up visit (*n* = 1,550), not enough follow up of HTN (*n* = 118), and cancer (*n* = 1) were excluded. Finally, data from 2,404 women were analyzed. Figure 1 shows the study participant’s selection process.

**Figure 2.**
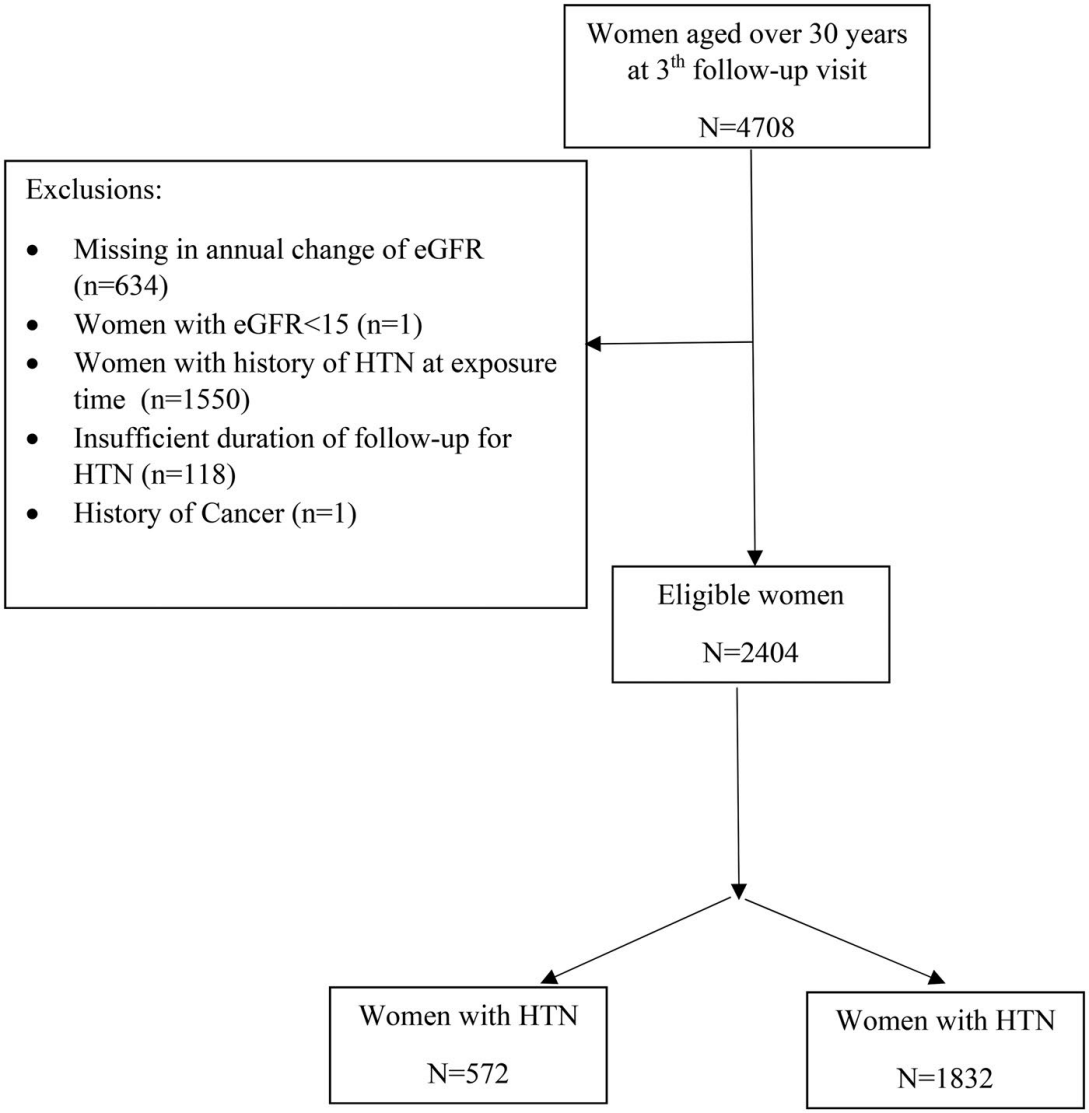
Flowchart of study.

Figure 2 demonstrates the flow of study variable measurements as follows:

**Figure 3.**
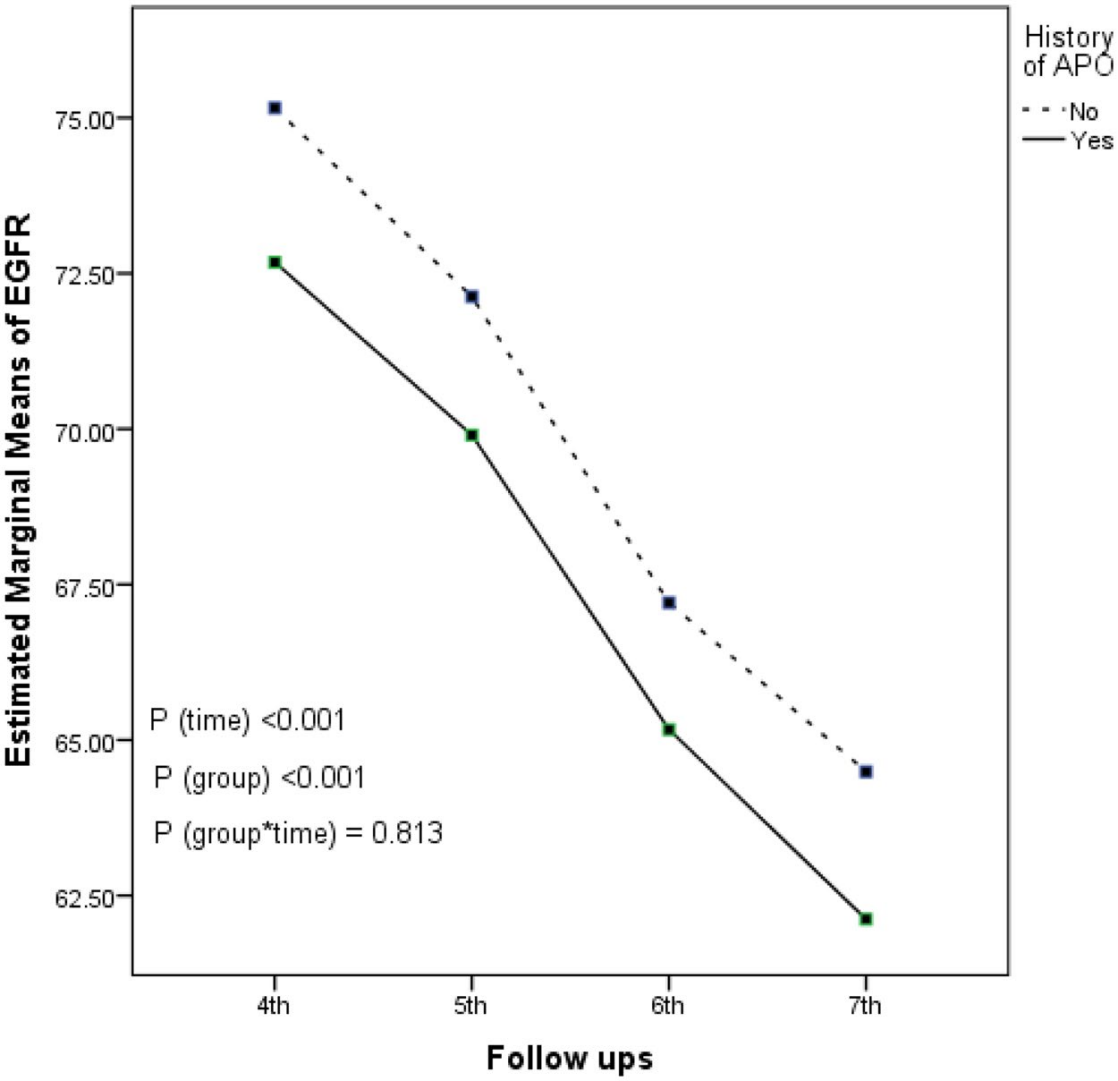
Longitudinal trends in eGFR stratified by history of adverse pregnancy outcomes (APOs) across follow-up visits.

**Exposure period**: Baseline, second, and third follow-up visits

- APO status: Define composite variable, including history of one or more of APOs including preterm birth, preeclampsia, gestational diabetes, and abortion/stillbirth in baseline, second and third follow-up visits.
- eGFR: Calculating annual change in eGFR in second and third follow-up visits

**Index phase**: Third follow-up visit (This phase is the starting point for follow-up)
**Follow-up period**: 4th, 5th, 6th follow-up visits for the experience of the event (HTN) or the end of follow-up without an experienced event.

The study protocol was approved by the ethics committee of the Research Institute for Endocrine Sciences, Shahid Beheshti University of Medical Sciences, and written informed consent was obtained from all participants (IR.SBMU.ENDOCRINE.REC.1403.102).

### Measurements

For the aim of demographic and clinical measurements, a trained interviewer performed data collection *via* questionnaire on demographic, family history of disease, drug history, and habitual status information. The modifiable activity questionnaire was applied to assess physical activity. The reliability and validity of the Persian version of the MAQ have been reported [26].

Blood pressure was evaluated after participants rested in a sitting position for 15 min. A standardized mercury sphygmomanometer (calibrated by the Iranian Institute of Standards and Industrial Research) was used to assess blood pressure on the right arm twice. For each participant, the average of the two measurements was recorded. Weight was measured using a digital scale (Seca 707; range: 0–150 kg; Seca GmbH, Germany, accuracy of 100 g), minimally clothed without shoes. Height was recorded in a standing position, with shoulders in neutral alignment, without shoes using a stadiometer (Seca 225; Seca GmbH, Germany, accuracy nearest 0.5 cm). Body mass index (BMI) was calculated as weight (kg) divided by the square of height (m^2^).

For biochemical measurements, for each participant in each phase, an overnight fasting (12–14 h fasting) venous blood sample was collected in a vacutainer tube, then centrifuged within 30–45 min of collection. Serum creatinine (Cr) levels were assayed by the kinetic colorimetric Jaffe method. The sensitivity of the assay was 0.2 mg/dL (range, 18 to 1,330 μmol/L [0.2 to 15 mg/dL]). Also, enzymatic colorimetric method was used for measuring of Total Cholesterol (TC), Triglycerides (TG), and HDL Cholesterol (HDL-C). Intra- and inter-assay coefficients of variation were both 3.0% for TC, 2.3% for TG, 4.6% for HDL-C, and 2.1% for creatinine.

Analyses were performed using Pars Azmon kits (Pars Azmon Inc, Tehran, Iran) and a Selectra 2 auto-analyzer (Vital Scientific, Spankeren, the Netherlands).

### Definition of HTN, APOs, and eGFR

Criteria for the diagnosis of hypertension were systolic blood pressure (SBP) above 140 mmHg or diastolic blood pressure (DBP) above 90-mmHg or drug use [27].

Adverse pregnancy outcomes in the current study includes history of preterm delivery, abortion, stillbirth, gestational diabetes (GDM), and pregnancy-induced hypertension/preeclampsia (PIH/PEC). Abortion was defined as pregnancy loss < 20 weeks of gestational age (GA) [28]. Stillbirth was defined the death of a fetus after gestational age of 22 weeks [29]. Preterm delivery was defined as delivery before 37 weeks of GA [30]. PIH was defined as SBP ≥140 mm Hg and/or a DBP ≥90 mm Hg on at least two occasions at least 6 h apart after the 20th week of GA in women known to be normotensive before pregnancy and before 20 weeks’ GA [31]. PEC was defined as blood pressure ≥140/90 mmHg, along with a protein excretion ≥0.3 g in a 24-h period after the 20th week of GA [32]. Moreover, GDM was defined according to the WHO criteria as the presence of any of the following criteria: fasting blood glucose ≥92 mg/dL, 1-h plasma glucose ≥180 mg/dL, and 2-h plasma glucose ≥153 mg/dL [33,34].

The following equation was used to calculate the estimated glomerular filtration rate 35]]:

≤0.7 creatinine level eGFR = eGFR= 141 x (Scr/0.7)-0.329 x 0.9929Age x 1.018
>0.7 creatinine level eGFR = eGFR= 141 x (Scr/0.7)-1.209 x 0.9929Age x 1.018
‘Scr’ is serum creatinine measured in mg/dl.

### Statistical analysis

Data are reported as median (25th and 75th percentiles) for continuous variables or percentages for categorical variables. Baseline characteristics were described across the event status, using the t-test and Chi-square test for continuous and categorical variables, respectively. The Mann–Whitney U test was applied for skewed and ordered variables. Cox proportional-hazards regression models were used to estimate the hazard ratios (HRs) and their 95% confidence intervals (CIs) for the incidence of HTN. The first model was crude, while the second model was adjusted for baseline age, BMI, physical activity, family history of HTN, HDL, TG, TC, SBP, and DBP, menopause age in subgroup of menopausal women. The interactions between APO and eGFR were assessed.

The equation for z-score calculation was as follows:

$$\begin{array}{c} z-\text{score }\left(x\right)=\left(x-\mu \right)/\sigma \text{ where x is a raw value},\\ \;\mu \;-\text{is the population mean }\left(\text{reference value}\right),\;\\ \text{and }\sigma \;-\text{ is the population standard deviation}.\;\\ \text{For example z}-\text{ score}.\text{ All statistical analyses were performed}\\ \text{ in STATA }\left(\text{version 12};\text{ STATA Inc}.,\text{ College Station},\text{ TX},\text{ USA}\right),\text{ and }\\ P-\text{values less than }0.0\text{5 were considered statistically significant}. \end{array}$$

## Results

During a follow-up period of 8.46 ± 2.43 years, 572 incident HTN cases were documented among 2,404 participants, accumulating 3459.88 patient-years of observations. Of total, 672 women were menopause at index phase. Table 1 presents the baseline clinical characteristics stratified by the HTN status. Compared with non-HTN ones, patients with HTN group were older, exhibited a higher BMI, and demonstrated a greater prevalence of a family history of HTN. Additionally, individuals with HTN had lower HDL levels compared to those without HTN (*p* < 0.05).

**Table 1.** Demographic and clinical characteristics of the study participants at index phase of study based on HTN event.

Variables		Total (Normotensive at baseline) *N* = 2,404	Final experienced event		*p*-value
			Healthy (*n* = 1,832)	HTN (*n* = 572)	
Age, year		43 (36–51)	41 (35–49)	50 (42.25-59)	<0.001
BMI, Kg/m^2^		27.99 (25.39–31.16)	27.53(24.97–30.44)	29.74 (27.11–32.89)	<0.001
Educational level, years, N (%)	<Diploma	1,916 (79.7)	1,407 (76.8%)	509 (89.0%)	<0.001
	> =Diploma	488 (20.3)	425(23.2%)	63 (11.0%)	
Marital status, N (%)	Not married	137 (5.7)	114 (6.2%)	23 (4.0%)	0.04
	Married	2,267 (94.3)	1,718 (93.8%)	549 (96.0%)	
Smoking, N (%)	No	2,274 (94.6)	1,734 (94.7)	540 (94.4)	0.821
	Yes	130 (5.4)	98 (5.3)	32 (5.6)	
Menopausal status, N (%)	No	1732 (72)	1,435 (78.3)	297 (51.9)	<0.001
	Yes	672 (28)	397 (21.7)	275 (48.1)	
Physical activity, N (%)	Moderate to high	702 (29.2)	559 (30.5%)	143 (25.0%)	0.01
	Low	1,702 (70.8)	1,273 (69.5%)	429 (75.0%)	
Family history of HTN, N (%)	No	2352 (97.8)	1,811 (98.9%)	541 (94.6%)	<0.001
	Yes	52 (2.2)	21 (1.1%)	31 (5.4%)	
HDL-C, mg/dl		51.51 (43–59)	51 (44–59)	49 (42–58)	0.02
TG, mg/dl		111 (80–159)	105 (76.25-148)	133.50 (100-184.75)	<0.001
TC, mg/dl		191 (168–216)	188 (166–213)	200 (176–224)	<0.001
SBP, mmHg		108.14 (100–116)	105 (99–112)	117 (110–124)	<0.001
DBP, mmHg		72 (68–79)	70 (67–78)	77 (70–81)	<0.001
APO history	No	1410 (58.7)	1,120 (61.1)	290 (50.7)	<0.001
	Yes	994 (41.3)	712 (38.9)	282 (49.3)	
eGFR (at index date) mL/min/1.73 m^2^		74.10 (12.56)	74.71 (67.11–83.13)	70.27 (13.25)	<0.001
eGFR annual change		−.007 (-.036-.030)	−.007 (-.0359-.0297)	−.007 (-.038-.033)	0.74

**Median (interquartile range).

Abbreviations: BMI, body mass index; HTN, hypertension; HDL-C, high-density lipoprotein cholesterol, TC: Total Cholesterol; TG, triglyceride; SBP, systolic blood pressure; DBP, diastolic blood pressure; APO, adverse pregnancy outcome; eGFR; estimated glomerular filtration rate.

Crude regression models revealed no significant association between the Z score of annual eGFR change (measured at the 2nd and 3rd follow-up visits) and HTN risk in the total population or in subgroups stratified by a history of APOs.

In adjusted model, a one standard deviation increase in annual eGFR change was associated with lower HTN risk in women with a history of APOs (HR = 0.816, 95% CI: 0.708–0.939, *p* = 0.005). Conversely, no significant association was detected among women without a history of APOs (HR = 1.027, 95% CI: 0.913–1.156, *p* = 0.645) (Table 2). Moreover, sub-group analysis among menopausal women at index phase with a history of APOs showed that annual eGFR change was associated with lower HTN risk (HR = 0.818, 95% CI: 0.671–0.996, *p* = 0.046).

**Table 2.** Hazard ratios (95% CI) for hypertension incidence associated with annual eGFR change in the overall population and stratified by history of adverse pregnancy outcomes (APOs).

Population	Models	Variables	Total		Women without APO		Women with APO	
			HR (95% CI)	P-value	HR (95% CI)	P-value	HR (95% CI)	P-value
Total women	Crude	Z Score of eGFR annual change	1.00 (0.92–1.08)	0.99	1.05 (.95-1.17)	0.28	0.92 (0.81–1.04)	0.19
	Adjusted	Z Score of eGFR annual change	0.933 (0.852–1.021)	0.131	1.027 (0.913–1.156)	0.654	0.816 (0.708–0.939)	0.005
		eGFR (at index date) mL/min/1.73 m^2^	1.005 (0.996–1.014)	0.290	1.001 (0.989–1.013)	0.861	1.010 (0.995–1.025)	0.203
		Age, year	1.043 (1.032–1.053)	<0.001	1.041 (1.027–1.055)	<0.001	1.044(1.028–1.061)	<0.001
		BMI, Kg/m^2^	1.040 (1.022–1.059)	<0.001	1.039 (1.015–1.064)	0.001	1.043 (1.014–1.074)	0.003
		Physical activity (reference: low)	0.830 (0.685–1.006)	0.057	0.819 (0.634–1.057)	0.126	0.827 (0.617–1.107)	0.202
		Family history of HTN(reference: No)	1.752 (1.210–2.537)	0.003	1.716 (0.952–3.091)	0.072	1.819 (1.123–2.945)	0.015
		HDL-C, mg/dl	0.998 (0.990–1.005)	0.554	1.003 (0.993–1.013)	0.548	0.990 (0.979–1.002)	0.105
		TC, mg/dl	0.999 (0.997–1.001)	0.339	0.999 (0.996–1.002)	0.575	0.999 (0.995–1.002)	0.420
		SBP, mmHg	1.055 (1.045–1.066)	<0.001	1.055 (1.042–1.069)	<0.001	1.057 (1.042–1.072)	<0.001
		DBP, mmHg	1.028 (1.014–1.042)	<0.001	1.027 (1.009–1.046)	0.004	1.030 (1.008–1.052)	0.006
Menopause subgroup	Crude	Z Score of eGFR annual change	0.886 (0.165,2.043)	0.928	0.884 (0.570–1.118)	0.255	1.045 (0.922–1.284)	0.124
	Adjusted	Z Score of eGFR annual change	0.940 (0.825–1.070)	0.351	1.091 (0.909–1.311)	0.349	0.818 (0.671–0.996)	0.046
		eGFR (at index date) mL/min/1.73 m^2^	0.994 (0.979–1.008)	0.410	0.986 (0.966–1.007)	0.186	0.997 (0.975–1.019)	0.782
		Age, year	1.029 (1.009–1.050)	0.005	1.031 (1.006–1.058)	0.016	1.020 (0.986–1.056)	0.244
		BMI, Kg/m^2^	1.018 (0.989–1.048)	0.234	1.022 (0.982–1.064)	0.279	1.011 (0.968–1.055)	0.630
		Physical activity (reference: low)	0.844 (0.638–1.116)	0.234	0.828 (0.558–1.228)	0.348	0.811 (0.540–1.217)	0.311
		Family history of HTN(reference: No)	1.662 (1.017–2.716)	0.042	1.585 (0.679–3.698)	0.287	1.845 (0.992–3.429)	0.053
		HDL-C, mg/dl	0.998 (0.987–1.008)	0.682	1.014 (1.000–1.028)	0.059	0.980 (0.964–0.996)	0.017
		TC, mg/dl	0.998 (0.995–1.001)	0.104	0.998 (0.994–1.002)	0.332	0.997 (0.993–1.002)	0.211
		SBP, mmHg	1.040 (1.027–1.054)	<0.001	1.036 (1.018–1.055)	<0.001	1.042 (1.023–1.062)	<0.001
		DBP, mmHg	1.018 (0.998–1.039)	0.076	1.026 (0.997–1.056)	0.081	1.013 (0.985–1.042)	0.366
		Menopausal age, year	1.004 (0.981–1.028)	0.747	1.001 (0.972–1.031)	0.931	1.021 (0.981–1.062)	0.319

Abbreviations: BMI, body mass index; HTN, hypertension; HDL-C, high-density lipoprotein cholesterol; TC: Total Cholesterol; APO; adverse pregnancy outcome; eGFR; estimated glomerular filtration rate; SBP: systolic blood pressure; DBP: diastolic blood pressure.

In Cox models, we observed a statistically significant interaction between APO status and annual eGFR change (interaction *p* = 0.02) (Table 3).

**Table 3.** Hazard ratios (95% CI) for hypertension incidence associated with interaction of annual eGFR change and history of adverse pregnancy outcomes (APOs)in the overall population and menopausal women.

Population	Models	Variables	HR (95% CI)	P-value
Total women	Crude	Z Score of eGFR annual change*APO	0.871 (0.740–1.025)	0.096
	Adjusted^⁕^	Z Score of eGFR annual change*APO	0.822 (0.698–0.969)	0.020
Menopausal women	Crude	Z Score of eGFR annual change*APO	0.801 (0.632–1.015)	0.067
	Adjusted^⁕⁕^	Z Score of eGFR annual change*APO	0.809 (0.641–1.019)	0.072

**^⁕^**Adjusted for age, body mass index; eGFR at index phase, family history of hypertension, high-density lipoprotein cholesterol, physical activity total cholesterol, systolic and diastolic blood pressure.

**^⁕⁕^**Adjusted for age, body mass index; eGFR at index phase, family history of hypertension, high-density lipoprotein cholesterol, total cholesterol, systolic and diastolic blood pressure, physical activity menopausal age.

We further assessed longitudinal trend in eGFR across follow-up visits and compared them between participants with and without APO history using GEE model. The analysis demonstrated that mean eGFR values were significantly lower in women with a history of APOs women compared to those without (mean difference: −3.44 units; 95% CI: 4.54, −2.35; *p* < 0.001). Furthermore, a significant declining trend in eGFR was observed across both groups, with a mean change of −3.70 units (95% CI: −3.87, −3.53; *p* < 0.001). No significant interaction effect was observed between APO history groups and eGFR trends over time (*p* = 0.478) indicating that the rate of eGFR change did not differ significantly between the two groups. Figure 3 illustrates the longitudinal trends of eGFR in participants stratified by APO history.

## Discussion

This study elucidates the complex interplay between a history of APOs, longitudinal changes in eGFR, and the subsequent risk of HTN in Iranian women who participated in a population-based cohort study. The key findings are as follows: 1) Women with a history of APOs demonstrated a consistently lower baseline eGFR compared to those without APOs. 2) A one-unit positive annual eGFR z-score change was associated with lower HTN risk among women with a history of APOs. This novel, clinically relevant finding underscores a critical link between time-dependent eGFR fluctuations and HTN risk in these high-risk women.

HTN remains the leading cause of cardiovascular mortality and morbidity worldwide, accounting for a substantial proportion of CVD burden and deaths [36], while traditional risk factors for HTN are well-established, sex-specific physiological and pathophysiological reproductive factors uniquely influence cardiovascular health in women [37]. Emerging evidence suggests that female reproductive health plays a pivotal role in the development of cardiovascular disease [38]. APO, such as preeclampsia, has been increasingly recognized as the important predictors of future cardiovascular risk. Notably, APOs are potentially associated with an accelerated decline in renal function, as measured by eGFR, which itself is closely linked to the development of HTN [39]. Women with a previous history of APOs, even at a relatively young age, may exhibit lower renal function compared to their counterparts without such a history, underscoring the long-term impact of reproductive health on cardiovascular and renal outcomes [40].

While healthy pregnant women experience physiologically increasing GFR levels up to 40–50% in early pregnancy, in women with complicated pregnancy this adaptation is often disrupted [41]. APOs are increasingly recognized not merely as transient obstetric complications but as early indicators of latent vascular and renal dysfunction, with long-term implications for cardiovascular health [42,43]. While traditional risk factors (e.g., obesity, sedentary lifestyle, and genetic predisposition) fail to account for a significant proportion of HTN cases, identifying novel, sex-specific risk factors—particularly those linked to reproductive health—remains a critical priority in cardiovascular epidemiology. A key unresolved question is whether APOs predispose women to subclinical declines in eGFR over time and whether such renal functional changes mediate the elevated HTN risk observed in this population.

Our findings substantiate the hypothesis that women with a history of APOs exhibited consistently lower eGFR values compared to their counterparts without APOs across all follow-up assessments. Specifically, women with APO exhibited an initial and sustained reduction in renal function relative to those without such a history. This progressive decline overtime may contribute to the underlying mechanisms of HTN pathogenesis. Turin et al. (2016), in a study involving 479,126 adults without a prior history of cardiovascular disease who underwent at least 3 eGFR measurements in Alberta, showed that individuals experiencing the greatest rate of eGFR decline (≤-5 mL/min/1.73 m2/year) had more than a twofold increased risk of cardiovascular events [44]. The observed decline in eGFR among women with APOs aligns with previous literature indicating that complicated pregnancies may unmask underlying vascular vulnerabilities [45]. Previous robust evidence also supported the elevated risk of long-term kidney disease in women with a history of complicated pregnancies [19].

In the present study, we observed that among women with a history of APOs, positive annual changes in renal function may confer a protective effect against HTN within this cohort. Conversely, among women without APOs, annual eGFR changes were not significantly associated with HTN risk. Positive annual change of eGFR may reflect lower systemic inflammation and better metabolic regulation, which in turn could reduce HTN risk. Additionally, high-risk perceptions among women with history of APOs may improve their lifestyle behaviors to mitigate future cardio-metabolic risk [46].

Findings showed that in postmenopausal women, the direction and magnitude of the association were consistent with those observed in the total population. Specifically, a positive annual eGFR change among women with a history of APO was associated with a lower risk of incident HTN.

The mechanism underlying this association may be multifactorial. It is proposed that kidney dysfunction is closely linked to endothelial damage, inflammation, and the immune system [47], all of which play important roles in the development of HTN [48]. Numerous pathways also contribute to mechanisms of linking APO to cardiovascular events including the development of a pro-inflammatory and antiangiogenic balance, cardiac structural and functional abnormalities following an APO, vascular and renal dysfunction during and after an APO, and behavioral factors [49]. Pregnancy is known as a physiological stress test for women, as the mother’s body’s adaptation to the conditions of pregnancy may predispose to disease, which may remain hidden for many years [50]. It is proposed as a golden opportunity for screening tests for subsequent cardiovascular events in life [51]. Postpartum prevention strategies to prevent or delay the early-onset of disease among women with a history of APOs provide a golden opportunity for optimal future maternal health [52]. Interestingly, the added value of obstetric information on risk prediction tools of CVD or CKD has been explored in the previous literature [53]. A detailed history of pregnancy complications is critical in preventive health screening [24]. Recent accumulating evidence underscores the critical role of pregnancy as a determinant in the subsequent risk of developing cardiometabolic disorders [10,49,54]. Moreover, longitudinal trajectories of eGFR have been demonstrated to serve as the significant predictors of CVD onset and progression [55]. Pregnancy thus represents a unique physiological window that not only reflects immediate maternal health but also provides valuable prognostic insight into long-term renal function and maternal kidney health [56].

This study findings should be interpreted in the context of some limitations and strengths. This study benefits from a large, community-based cohort, which enhances the generalizability of the results to the broader population. The use of standardized protocols for data collection ensured consistency and reliability across measurements. Additionally, the application of rigorous longitudinal analyses allowed for a comprehensive assessment of temporal changes and their associations with clinical outcomes. Importantly, we utilized the most up-to-date and validated formula for estimating the eGFR, thereby improving the accuracy of renal function assessment. Despite these strengths, several limitations warrant consideration. First, the exclusion of participants with missing data raises the possibility of selection bias, which could compromise the representativeness of the study sample. However, this concern is partially mitigated by the lack of statistically or clinically significant differences in key baseline characteristics (e.g., age, BMI, or cardiometabolic profiles) between excluded individuals and those retained in the analysis. Second, our reliance on serum creatinine-based eGFR estimates, while standard in clinical research, is subject to variability influenced by non-renal factors such as muscle mass, diet, and medication use, potentially impacting the precision of renal function measurement. Third, the observational design of the study inherently limits causal inference, precluding definitive conclusions regarding the directionality of the observed associations. Although stratification by APO subtype would provide more granular insights, the number of participants within individual APO categories in this study was insufficient to support statistically reliable subtype-specific analyses. So, the future larger, multicenter cohorts suggested to perform detailed APO subtype stratification to better characterize differential associations. Finally, although adjustments were made for a comprehensive set of available covariates, residual confounding by unmeasured or unknown factors cannot be entirely excluded, which may influence the associations reported herein.

Future research should focus on clarifying the causal mechanisms linking APOs, longitudinal renal function trajectories, and the subsequent development of HTN through prospective and interventional study designs. The use of more sensitive biomarkers of kidney function such as cystatin C and urinary albumin-to-creatinine ratio, along with inflammatory, endothelial, and vascular markers, may improve risk stratification and mechanistic understanding. Integrating multi-omics approaches including genomics, proteomics, and metabolomics could further identify biological pathways that predispose women with a history of APOs to long-term cardio-renal disease. In addition, randomized or pragmatic trials evaluating postpartum interventions, including structured renal surveillance, lifestyle modification, and early pharmacological prevention, are needed to determine whether improving or preserving eGFR trajectories can reduce the incidence of hypertension. Expanding such studies to diverse populations and incorporating reproductive history into cardiovascular and renal risk prediction models will support a life course and sex-specific approach to prevention and clinical care.

## Conclusion

In conclusion, this study provides novel evidence that a positive longitudinal change in eGFR among women with a history of APOs is associated with a reduced risk of HTN. These findings advance our understanding of the interplay between reproductive health, renal function dynamics, and cardiovascular risk in women.

## Data Availability

The data that support the findings of this study are available from the corresponding author, [F.R.T.], upon reasonable request.

## Abbreviations

APO: adverse pregnancy outcome
CVDs: cardiovascular diseases
SBP: systolic blood pressure
DBP: diastolic blood pressure
TC: total cholesterol
TG: triglyceride
CI: confidence interval
GA: gestational age
GDM: gestational diabetes mellitus
PIH/PEC: pregnancy-induced hypertension/preeclampsia
PTD: preterm delivery
HTN: hypertension
eGFR: estimated glomerular filtration rate.
